# Adapting RISE Into Montana Adult Protective Services: An Embedded Model

**DOI:** 10.1093/geroni/igaf122.088

**Published:** 2025-12-31

**Authors:** David Burnes, Marian Liu, Patricia Kimball, Lianna Waller, Jody McCampbel, Marie-Therese Connolly, Geoff Rogers, Stuart Lewis

**Affiliations:** University of Toronto, Toronto, Ontario, Canada; Purdue University, West Lafayette, Indiana, United States; Elder Abuse Institute of Maine, Brunswick, Maine, United States; Adult Protective Services, Missoula, Montana, United States; Adult Protective Services, Billings, Montana, United States; RISE Collaborative, Washington, District of Columbia, United States; Hunter College, New York, New York, United States; Dartmouth Health, Hanover, New Hampshire, United States

## Abstract

Understanding effective elder abuse and self-neglect (EASN) interventions remains limited. Adult Protective Services (APS) is primarily responsible for investigating EASN reports in the U.S. However, APS generally lacks a dedicated intervention phase to address underlying case needs/risks. RISE is an evidence-based EASN intervention that works in partnership with APS to address this intervention gap. RISE integrates core modalities (motivational interviewing, restorative approaches, teaming, engagement, goal attainment scaling) and operates at Relational, Individual, Social, and Environmental levels to work with older adult victims and alleged harmers and strengthen social supports surrounding them. To date, the RISE-APS model has involved complementary partnerships where RISE operates from external community-based organizations. In partnership with Montana APS, this study sought to understand how to adapt and implement a RISE-APS “embedded” model, where RISE operates from within Montana APS as a specialized unit providing extended services for complex cases. This RISE-APS adaptation/implementation project was guided by the Consolidated Framework for Implementation Research (CFIR), containing factors across five domains (intervention, outer setting, inner setting, individuals, implementation process) representing potential internal/external implementation barriers/facilitators. Informed by CFIR and using a descriptive phenomenological qualitative approach, we conducted focus groups and individual interviews with Montana APS management (n = 4) and social service workers (5) as part of an adaptation needs assessment to identify implementation barriers/facilitators and engaged in a process to determine strategies to address/leverage them. This presentation will present an adaptation process model for the embedded RISE-APS model, identifying implementation determinants (barriers, facilitators), actions (strategies, mechanisms), and outcomes (adoption, fidelity, sustainment).

